# p53 amplifies Toll-like receptor 5 response in human primary and cancer cells through interaction with multiple signal transduction pathways

**DOI:** 10.18632/oncotarget.4435

**Published:** 2015-06-10

**Authors:** Maria Shatz, Igor Shats, Daniel Menendez, Michael A. Resnick

**Affiliations:** ^1^ Chromosome Stability Group, Laboratory of Molecular Genetics, National Institute of Environmental Health Sciences, NIH, Research Triangle Park, NC, USA; ^2^ Department of Biomedical Engineering, Duke University, Durham, NC, USA

**Keywords:** p53, Toll-like receptor 5, cancer, signal transduction, inflammation

## Abstract

The p53 tumor suppressor regulates transcription of genes associated with diverse cellular functions including apoptosis, growth arrest, DNA repair and differentiation. Recently, we established that p53 can modulate expression of Toll-like receptor (TLR) innate immunity genes but the degree of cross-talk between p53 and TLR pathways remained unclear. Here, using gene expression profiling we characterize the global effect of p53 on the TLR5-mediated transcription in MCF7 cells. We found that combined activation of p53 and TLR5 pathways synergistically increases expression of over 200 genes, mostly associated with immunity and inflammation. The synergy was observed in several human cancer cells and primary lymphocytes. The p53-dependent amplification of transcriptional response to TLR5 activation required expression of NFκB subunit p65 and was mediated by several molecular mechanisms including increased phosphorylation of p38 MAP kinase, PI3K and STAT3 signaling. Additionally, p53 induction increased cytokine expression in response to TNFα, another activator of NFκB and MAP kinase pathways, suggesting a broad interaction between p53 and these signaling pathways. The expression of many synergistically induced genes is elevated in breast cancer patients responsive to chemotherapy. We suggest that p53's capacity to enhance immune response could be exploited to increase antitumor immunity and to improve cancer treatment.

## INTRODUCTION

The p53 tumor suppressor functions as a sequence-specific transcription factor to regulate expression of genes associated with a wide range of cellular functions including apoptosis, cell cycle arrest, senescence, DNA repair, differentiation and glycolysis [1]. Recently, we demonstrated that induction of p53 protein in primary human leukocytes and in a variety of cancer cell lines induces expression of the innate immune Toll-like receptor (TLR) family genes. We also found that p53 modulates TLR expression in humans but not in rodents [2, 3].

The TLRs are membrane glycoproteins that recognize a variety of distinct pathogen-associated molecular patterns (PAMPs) [4]. Ten human TLRs (TLR1-10) are expressed in several types of immune cells including spleen, T and B lymphocytes, dendritic cells and macrophages [5], and in non-immune tissues, such as airway and epithelial cells that have direct contact with pathogens [6, 7]. TLR stimulation by ligands leads to activation of NFκB, interferon responsive factors and the mitogen-activated kinase (MAPK) pathway resulting in distinct expression patterns of immune/inflammatory genes required for pathogen elimination [8]. Deregulated expression and activity of TLRs are associated with excessive inflammation and inflammation-induced diseases including idiopathic inflammatory myopathies [9], multiple sclerosis [10] and rheumatoid arthritis [11]. Ligands for various TLRs have been used as adjuvants in cancer treatment in both experimental and clinical settings. For example, the TLR7 and TLR9 agonists are used to treat skin cancer and cutaneous T-cell lymphoma (reviewed in [12]), and TLR9 agonists are being tested in clinical trials against NSCLC [13]. High expression of Toll-like receptor 5 correlates with better prognosis in non-small-cell lung cancer [14]. A derivative of TLR5 ligand flagellin, CBLB502 demonstrated antitumor activity in mice and protected normal tissue from radiation-induced damage [15].

p53 is generally considered to repress inflammation [16]. Mutant p53 mice are highly prone to inflammation-associated colorectal cancer with increase in chronic NFκB activation, resulting in tissue damage and extended inflammation [17]. The specific deletion of p53 in intestinal epithelial cells causes upregulation of many inflammation and epithelial-mesenchymal transition-associated genes, creates an inflammatory microenvironment and increases incidence of carcinogen-induced tumors [18]. Basal levels of proinflammatory cytokines in the lungs of p53 knockout mice are elevated compared with wild type p53 animals [19].

On the other hand, several studies have demonstrated that under specific conditions p53 can actually promote inflammation. Upregulation of p53 in adipose tissue increases inflammation through accelerated lipolysis, while disruption of p53 activation attenuates inflammation [20]. Similarly, p53 deficiency in a rat model mitigates carcinogen-induced hepatic inflammation, cirrhosis, and tumorigenesis by attenuating release of HMGB1, the major damage-associated alarmin [21]. A polymorphism at p53 codon 72 can affect the response to inflammatory challenge, where the P72 variant enhances cytokine response in thymocytes from LPS-treated animals [22]. Also, short term (2 hours) activation of p53 by Nutlin-3 [23] in human macrophages induces inflammation-like transcriptional response [24].

Finally, as mentioned above, p53 can increase expression of TLRs and the transcriptional response induced by their activation, which may result in increased inflammation [2, 3, 25]. Based on this observation, we decided to examine the genome-wide effect of p53 induction by Nutlin-3 on the transcriptional response to the TLR5 ligand flagellin. We identified a large group of genes with expression amplified by combined treatment as compared to either single agent. Moreover, we discovered several molecular mechanisms that could account for enhancement of the TLR5 pathway activity by p53.

## RESULTS

### The TLR5-mediated gene expression program is greatly enhanced by p53

Recently, we showed that induction of p53 protein using doxorubicin, 5-fluorouracil, UV, ionizing radiation and MDM2 inhibitor Nutlin-3 can modify expression of TLRs and enhance expression of IL1B, IL6 and IL8 cytokines in response to TLR ligands in human primary blood cells and cancer cell lines [2, 3]. Therefore, we decided to study systematically the genome-wide effects of p53 on TLR-mediated gene expression. We focused on TLR5 signaling in MCF7 breast adenocarcinoma cells, since TLR5 is highly expressed and is generally responsive in breast cancer cells.

**Figure 1:**
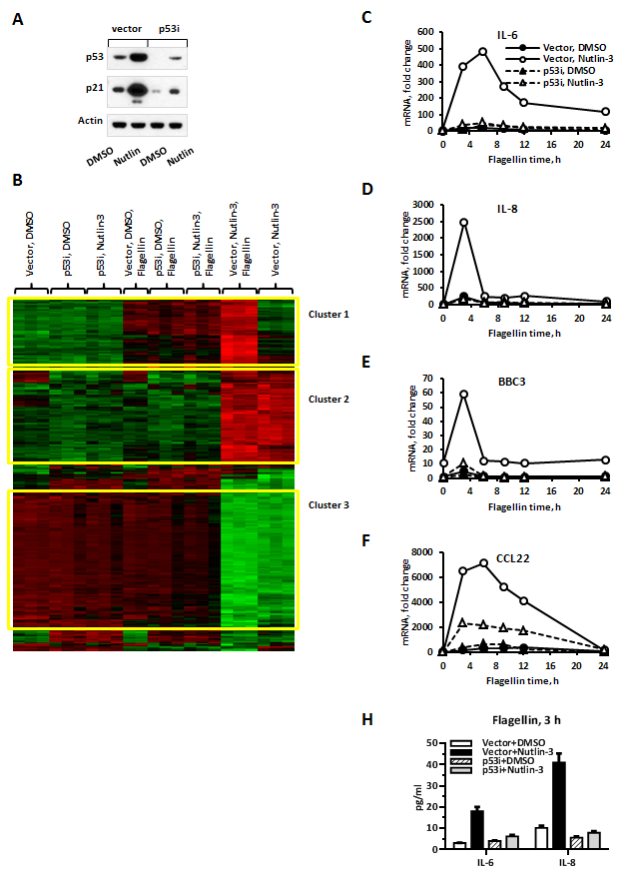
TLR5-mediated gene expression is greatly facilitated by p53 induction in MCF7 cells **A.** MCF7-vector or MCF7-p53i cells were treated for 48 h with Nutlin-3 (10 μM) or vehicle control DMSO (0.1%). Following the treatment the cells were harvested and protein levels of p53 and p21 were assessed by Western blot. **B.** Genome-wide gene expression in MCF7-vector or MCF7-p53i cells treated with Nutlin-3 or DMSO for 48 h followed by an additional 3 h with flagellin (500 ng/ml) was examined using Affymetrix microarrays. Presented is hierarchical clustering visualized using Java TreeView. Each replicate represents individual sample from independent experiment. **C.**-**F.** MCF7-vector or MCF7-p53i cells were incubated with Nutlin-3 or DMSO for 48 h. During the last 24 h the medium was replaced with DMSO/Nutlin-3 medium containing flagellin for indicated times. All the samples were harvested at the same time. mRNA was purified and IL-6, IL-8, BBC3 and CCL22 gene expression was assessed using pre-designed real time PCR assays. **H**. Cells were treated as described in C. and concentrations of IL-6 and IL-8 protein in the supernatant were assessed using a Bioplex assay. qPCR experiments were repeated 2-6 times; presented is a representative experiment with samples run in duplicates. Bars indicate range for PCR replicates.

MCF7 cells expressing wild type p53 stably transfected with control empty vector (referred to as “vector” cells) or with shRNA targeting p53 (referred to as “p53i” cells) were treated with either Nutlin-3 or the vehicle control DMSO for 48 h. The treatment increased protein levels of p53 and its well-established target p21 in a p53-dependent manner (Figure 1A). Next, cells were exposed to the TLR5 ligand flagellin for 3 h, harvested and submitted to microarray-based gene expression analysis. Hierarchical clustering of the most differentially expressed probes revealed 3 major clusters. One large cluster (Figure 1B, Cluster 2) was characterized by specific upregulation in MCF7-vector cells treated with Nutlin-3 alone or in combination with flagellin. Thus, Cluster 2 represents genes upregulated by activated p53. Importantly, lack of induction of this large cluster in MCF7-p53i cells demonstrates the efficiency of functional p53 inactivation by shRNA and further validates the fact that Nutlin-3 effects are mediated specifically through p53 activation. Multiple well-established p53 targets such as *BAX*, *CDKN1A* (p21), *CCNG2* (cyclin G2), *GADD45*, *MDM2*, *SERPINB5* (Maspin) and *SESN1/2* (Sestrin1/2) were found among the 259 genes in this cluster. Consistent with known p53 functions as an inducer of cell cycle arrest and apoptosis, the top functional Gene Ontology (GO) categories in Cluster 2 were Regulation of Cellular Physiological Processes, Regulation of Cell Cycle and Induction of Programmed Cell Death (Table 1).

**Table 1:** Gene Ontology (GO) for genes with expression altered by Nutlin-3 or by flagellin

Gene Ontology (genes#; *p*-value<)		
Flagellin induced genes (199)	Nutlin-3 induced genes (259)	Nutlin-3 repressed genes (339)
immune response (79; 0.0001)	regulation of cellular physiological processes (23; 0.0001)	cell cycle; (309; 0.0001) DNA replication (149; 0.0001)
response to biotic stimulus (90; 0.0001)	regulation of cell cycle (18; 0.0001)	DNA replication and chromosome cycle (182; 0.0001)
defense response (85; 0.0001)	cell proliferation (32; 0.0002)	cell proliferation (328; 0.0001)
response to external biotic stimulus (59; 0.0001)	induction of programmed cell death (9; 0.0003)	DNA metabolism (206; 0.0001)
response to wounding (45; 0.0001)	induction of apoptosis (9; 0.0003)	M phase (110; 0.0001)
inflammatory response (37; 0.0001)		nuclear division (103; 0.0001)
**KEGG pathway:** RIG-I-like receptor signaling pathway; Cytokine-cytokine receptor interaction; Toll-like receptor signaling pathway		mitotic cell cycle (107; 0.0001)
	**KEGG pathway:** p53 signaling pathway	**KEGG pathway:** cell cycle

Gene clusters identified by hierarchical clustering as Flagellin induced (Cluster 1), Nutlin-3 induced (Cluster 2) and Nutlin-3 repressed (Cluster 3) were analyzed using Gather interface to detect statistically enriched GO terms.

An additional large cluster of 339 genes (Figure 1B, Cluster 3) was downregulated by Nutlin-3 in MCF7-vector cells in a p53 specific manner. This cluster was highly enriched for GO categories Cell Cycle, Cell Proliferation and included numerous genes identified with cell cycle progression such as cyclins A, B and E2, multiple cell division cycle (CDC) and minichromosome maintenance (MCM) genes and DNA polymerases (Table 1). The promoters of Cluster 3 genes were dramatically enriched for E2F binding sites (*p* < 0.0001) consistent with indirect repression of this cluster through the p53/p21/E2F axis [29, 30]. Another large cluster contained 199 genes induced specifically by flagellin. The flagellin-induced gene expression profile was similar in MCF7-vector and in MCF7-p53i cells (Figure 1B, Cluster 1). Most of these genes fall into the Innate Immune Response category (Table 1) and are enriched for NFκB binding sites (*p* < 0.0001).

Intriguingly, the global transcriptional response to flagellin was significantly amplified if the MCF7-vector cells were pretreated with Nutlin-3 (Figure 1B). We identified 208 annotated genes, designated as Synergistic Targets (Suppl. Table 1), whose expression was synergistically increased by the combination of Nutlin-3 and flagellin in a p53-dependent manner as compared to either Nutlin-3 or flagellin alone (Cluster 1, Vector + Nutlin-3 + Flagellin; see Material and Methods for definition of synergy). This gene set includes chemokines and cytokines (*IL6, IL8, CCL20, CCL22, CXCL11*), antimicrobial proteins (*GBP1, GBP2*), inflammation-related genes (*ATF3, S100A7-9, PLAU, PLAUR, WNT4, UBD*) and apoptosis regulating genes (*BBC3, BCL2A1, BIK*). A KEGG pathway analysis demonstrated significant enrichment for TLR receptor, cytokine-cytokine receptor interaction and JAK-STAT signaling pathways. An analysis of transcription factor binding sites identified strong enrichment for NFκB response elements in the promoter area of these genes suggesting that Nutlin-3 enhances NFκB-mediated TLR5 signaling (Table 2). To confirm that the synergistic gene expression is TLR5-dependent we performed siRNA-mediated TLR5 knockdown and found that it prevented flagellin-mediated transcriptional response (Suppl. Figure 1).

**Table 2:** Gene annotations for Synergistic Targets

Gene Ontology (# of genes; *p*-value<)	Transcription factors (# of genes; *p*-value<)
Immune response (24; 0.0001)	NF-kappaB (47; 0.0001)
Response to external biotic stimulus (19; 0.0001)	Ikaros 3 (31; 0.0001)
Response to biotic stimulus (27; 0.0001)	**KEGG pathway (genes#; *p*-value<)**
Defense response (25; 0.0001)	Toll-like receptor signaling pathway (7; 0.0003)
Response to pest, pathogen or parasite (18; 0.0001)	Cytokine-cytokine receptor interaction (11; 0.0005)
Inflammatory response (11; 0.0001)	Jak-STAT signaling pathway (8; 0.0009)

Genes identified as Synergistic Targets (see Materials and Methods and Suppl. Table 1) were analyzed using Gather interface to identify statistically over-represented GO terms, transcription factors binding elements in promoter areas of these genes and KEGG signaling pathways.

The kinetics of the synergistic increase in gene expression was examined by qPCR for several of the most responsive Synergistic Targets including *BBC3, IL6, IL8,* and *CCL22* (Figure 1C-1F). There was a dramatic, p53-specific increase in gene expression, mostly at early times (3-6 h), following flagellin addition in MCF7-vector cells pre-treated for 48 hours with Nutlin-3, ranging from approximately 3- to 12-fold. In addition, we analyzed IL6 and IL8 protein concentrations in supernatants of MCF7-vector or MCF7-p53i cells treated with Nutlin-3 or DMSO followed by 3 hours of flagellin treatment. The concentrations of IL6 and IL8 were 4-6-fold higher in supernatants collected from MCF7-vector cells that were exposed to Nutlin-3 prior to flagellin as compared to other conditions (Figure 1H). The synergistic increase in gene expression and p53 dependency was also consistent in additional Synergistic Targets including *ATF3, SAA2, PLAU, PLAUR, S100A8, S100A9, WNT4* and *UBD* (Suppl. Figure 2).

### p53 enhances ligand-dependent induction of inflammation genes in several cell types

**Figure 2:**
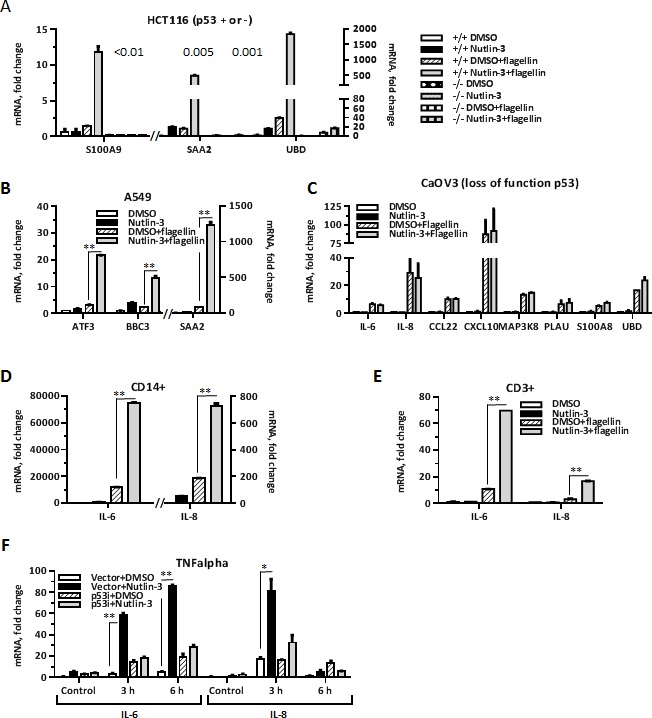
p53 enhances ligand-dependent expression of inflammation genes in several cell types **A.** HCT116 p53 ^+/+^ or HCT116 p53^−/−^ cells and **B.** A549 cells were incubated with Nutlin-3 (10 μM) or DMSO (0.1%) for 48 h. During the last 3 h the culture medium was replaced with DMSO/Nutlin-3 medium containing 500 ng/ml flagellin. After harvesting, the mRNA was purified and expression of several genes identified earlier as Synergistic Targets was assessed using pre-designed real time PCR assays. Shown is a representative experiment repeated 3 times. Bars indicate range for technical PCR replicates. **C.** CaOV3 cells were incubated with Nutlin-3 or DMSO for 48 h. During the last 3 h the medium was replaced with DMSO/Nutlin-3 medium containing flagellin for 3 h. The mRNA was purified and expression of indicated Synergistic Targets genes was assessed using pre-designed real time PCR assays. Shown is a representative experiment repeated twice. Bars indicate range for technical PCR replicates. **D.**-**E.** Freshly isolated human CD14^+^ cells and CD3^+^ cells, respectively, were incubated with Nutlin-3 or DMSO for 24 h. During the last 3 h the culture medium was replaced with DMSO/Nutlin-3 medium containing 500 ng/ml of flagellin. mRNA expression of IL6 and IL8 was measured by qPCR. Shown is a representative experiment (cells collected from one individual) repeated twice. The bars indicate range for technical PCR replicates. **F.** MCF7-vector or MCF7-p53i cells treated with Nutlin-3 or DMSO for 48 h were incubated with or without TNFα (10 ng/ml) for 3 or 6 h. Cells were harvested and mRNA expression of IL6 and IL8 was measured by qPCR. Shown is a representative experiment repeated 3 times. The bars indicate range for PCR replicates. *p < 0.05, **p < 0.01; Student's *t*-test was performed using GraphPad Prizm software.

To determine whether there is a direct connection between increase in receptor expression and increased response to ligand, we used HCT116 human colorectal carcinoma and A549 lung adenocarcinoma cells wherein Nutlin-3 mediated stabilization of p53 protein does not increase the level of TLR5 [2]. We still found that treatment with Nutlin-3 greatly enhanced flagellin-induced expression of several Synergistic Targets including *ATF3, BBC3, SAA2, S100A9* and *UBD* in these cells (Figure 2A and 2B). Contrary to that, the synergy was not observed in HCT116 p53^−/−^ colorectal carcinoma (Figure 2A) or in Caov3 ovary adenocarcinoma cells (Figure 2C) expressing functional TLR5 and a loss-of-function p53 mutant, demonstrating a requirement for transcriptionally active p53. We also found that the amplification is not specific to cancer cells but can occur in primary T-lymphocytes (CD3+ cells) or monocytes (CD14+ cells) freshly isolated from human blood and treated with Nutlin-3 for 24 hours prior to flagellin exposure, as shown by the increase in IL6 and IL8 expression in Figure 2D and 2E. In addition, we found that p53-mediated amplification of cytokine response extends beyond the TLR pathway. Nutlin-3 treatment of MCF7-vector cells led to an increase in IL6 and IL8 expression in cells exposed to TNFα (Figure 2F). The TNFα receptor levels were not induced by Nutlin-3 in the MCF7 cells (as indicated by microarray data).

Collectively, our results demonstrate that p53 can enhance ligand-dependent induction of multiple inflammation genes in a wide range of cell types in response to diverse stimuli without a requirement for increase in ligand receptor.

### p65 is required for the p53 enhancement of flagellin-induced gene expression

**Figure 3:**
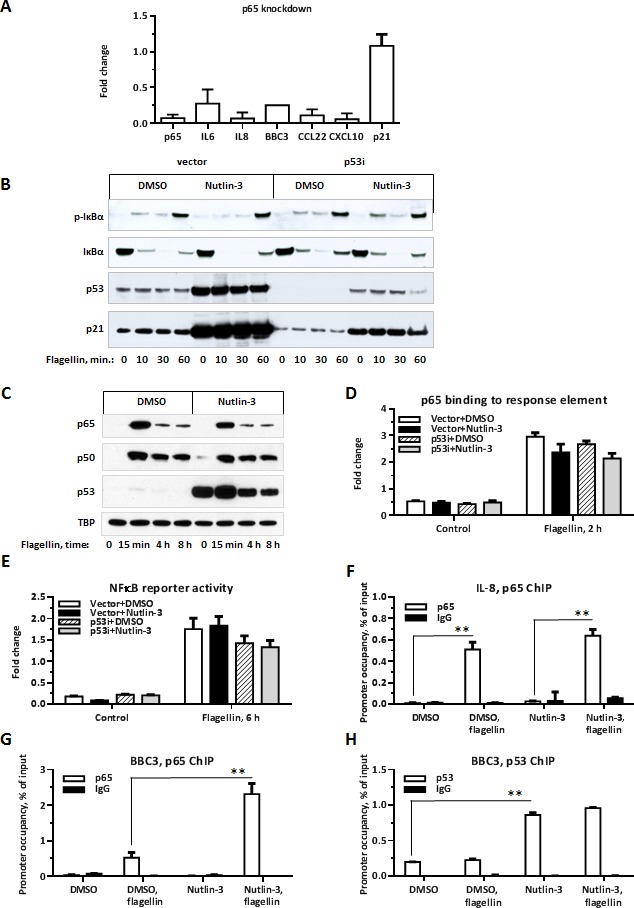
p65 is required for p53-enhancement of flagellin-induced gene expression **A.** MCF7-vector cells were transfected with p65 siRNA or control oligos (Dharmacon). 24 h later the cells were incubated with Nutlin-3 for additional 48 h and then exposed to flagellin for 3 h. Presented are fold-changes in mRNA expression for the indicated Synergistic Target genes in cells transfected with p65 siRNA relative to expression in cells transfected with control oligonucleotides. The experiment was repeated 3 times. **B.** MCF7-vector and MCF7-p53i cells treated with Nutlin-3 or DMSO for 48 h were incubated with flagellin for the indicated times. Cells were harvested and subjected to SDS-PAGE followed by Western blot analysis. Activation of NFκB pathway was assessed through phosphorylation and degradation of IκBα. The experiment was repeated 3 times. **C.** MCF7-vector cells treated with Nutlin-3 or DMSO for 48 h were incubated with flagellin for indicated times. Cells were harvested and subjected to subcellular fractionation. 15 μg of nuclear extracts were resolved by SDS-PAGE and then analyzed by Western blot to examine p65, p50 and p53 nuclear translocation. TBP was used as a control for fractionation and equal loading. The experiment was repeated 3 times. **D.** MCF7-vector or MCF7-p53i cells were treated with Nutlin-3 or DMSO for 48 h and then incubated with flagellin for 2 h. Cells were lysed and 5μg of total protein was used to measure p65 *in vitro* binding to immobilized oligonucleotides containing NFkB consensus binding site (Active Motif, TransAM p65). The experiment was repeated twice. **E.** MCF7-vector cells were co-transfected with pNFκB-Luc vector (240 ng/well, Clontech) and pRL-TK vector (10 ng/well, Promega), 24 h later cells were incubated with Nutlin-3 for 48 h and exposed to flagellin for 6 h. Cells were lysed and luciferase activity was measured using Dual Glo luciferase assay (Promega). Presented is an average of NFκB-driven luciferase activity normalized to Renilla luciferase activity for each sample. Each transfection was performed in triplicate and the experiment was repeated twice. **F.-H.** Cells were treated as in D and p65 occupancy at IL8 and BBC3 and p53 occupancy at BBC promoters were assessed by ChIP-qPCR. Each experiment was repeated twice. *p < 0.05, ** *p* < 0.01.

We addressed molecular mechanisms that might underlie the enhancement of TLR5 signaling by p53. Promoter analysis revealed a highly significant enrichment for NFκB transcription factor response element sequences in the promoter regions of Synergistic Targets (Table 2). NFκB is a major signal transduction pathway mediating TLR signaling [31] and, as expected, siRNA mediated knockdown of NFκB subunit p65 strongly reduced the induction of synergistic genes (Figure 3A). Therefore, we examined whether NFκB activation was modified by the increased p53 levels. The activation of the NFκB pathway in cells treated with flagellin was probed by phosphorylation and degradation status of IκBα. IκBα sequesters NFκB in the cytosol by masking its nuclear localization signal, thereby inhibiting its transcriptional activity [32]. Upon stimulation, IκBα is quickly phosphorylated and degraded allowing translocation of p65 to the nucleus where p65 transactivates target genes. IκBα was phosphorylated and degraded following 10-30 min incubation with flagellin (Figure 3B); however, these phosphorylation and degradation were not affected by p53 expression. To assess an involvement of multiple NFκB subunits in an induction of Synergistic Targets we incubated MCF7 cells for an hour with a pan-inhibitor of NFκB activity Bay-117082 prior to exposure to flagellin. Bay-117082 completely blocked induction of IL6 and IL8 as shown in Suppl. Figure 3. Of notice, a combination of Nutlin-3 and Bay-117082 in either presence or absence of flagellin reproducibly caused rapid cell death suggesting that NFκB activity is essential to overcome p53-induced cellular toxicity.

Next, we examined the possibility that p53 enhances the nuclear translocation and NFκB transcriptional activity. Nuclear translocation of NFκB p65 and p50 subunits following incubation with flagellin was comparable between cells treated with either DMSO or Nutlin-3 (Figure 3C). The rapid and transient kinetics of NFκB nuclear translocation were consistent with previous reports [33]. The *in vitro* binding of p65 to synthetic oligonucleotides containing NFkB consensus binding site has increased ~5-fold after flagellin, but it was not modified by the presence of Nutlin-3 (Figure 3D). In addition, the activity of NFκB-driven luciferase reporter following 6 h of flagellin treatment was not enhanced by p53 (Figure 3E).

We also compared the effect of p53 stabilization on the recruitment of p65 to promoters with and without p53 binding sites using chromatin immunoprecipitation. The recruitment of p65 to IL8 promoter (no p53 RE) was increased over 20-fold by flagellin treatment, but was not affected by p53 induction (Figure 3F). However, 16-fold induction of p65 recruitment to the NFκB response element in the BBC3 promoter by flagellin was further enhanced 4-fold by Nutlin-3 (Figure 3G). BBC3 contains both NFκB and p53 response elements separated by ~1.7 kb in its promoter region. While initially identified as a p53 target with several well-described response elements, BBC3 was later shown to also be induced by TNFα via an NFκB response element [34, 35]. Our results demonstrate that BBC3 expression is responsive to TLR activation and that BBC3 is synergistically induced by p53 and TLR5 signaling.

We also tested the possibility that binding of p53 to the promoters of Synergistic Targets is increased in presence of flagellin and possibly drives enhanced transcription of these genes. We found that 89 of the Synergistic Targets are bound by p53 following 8 h Nutlin-3 treatment in MCF7 cells based on published ChIP-Seq results including genes BBC3, PLAU and ATF3 [36]. We chose several Synergistic Targets that have potential p53 binding site and/or are bound by p53 and performed p53 ChIP qPCR on these genes. We found that p53 binding to BBC3 promoter increases ~4.5-fold over control DMSO in presence of Nutlin-3 but no significant increase in binding was observed in the presence of flagellin (Figure 3H). Similarly, p53 binding to IL6, CCL20, PIK3R3 or CSF1 promoters was not further increased by combined treatment (Suppl. Figure 4).

### Flagellin-induced p38 signaling is amplified by p53 and controls expression of several Synergistic Target genes downstream of TLR5

**Figure 4:**
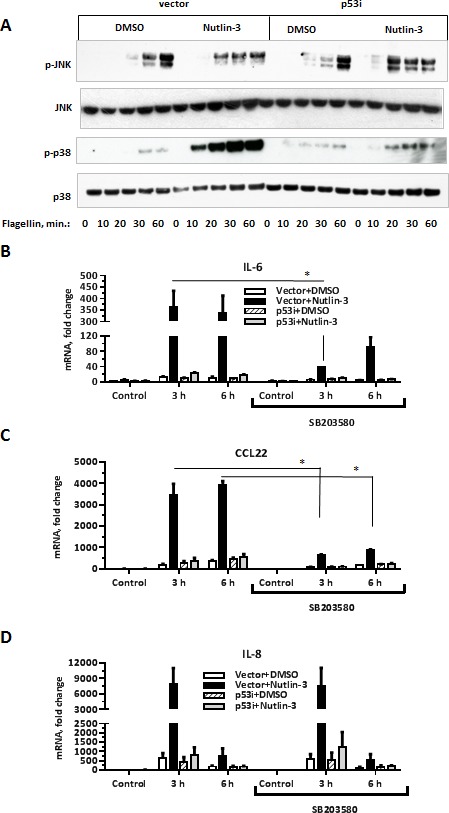
p53 activation specifically increases flagellin-induced phosphorylation of the p38 MAP kinase **A.** MCF7-vector or MCF7-p53i cells were incubated with Nutlin-3 or DMSO for 48 h. During the last 60 min, the medium was replaced with fresh DMSO/Nutlin-3 medium and the cells were incubated with flagellin (500 ng/ml) for the indicated times. Whole cell lysates were resolved on SDS-PAGE gel and then subjected to Western blot analysis to assess phosphorylation of p38 and JNK. Total p38 was used as a loading control. The experiment was repeated 3 times. **B.**-**D.** During the last 8 h of Nutlin-3 treatment the medium was replaced with DMSO/Nutlin-3 medium containing the p38 inhibitor SB203580 (10 μM) for 90 min and then incubated with flagellin for 3 h or 6 h. All the samples were harvested at the same time and IL-6, CCL22 and IL-8 gene expression was assessed by qPCR. The experiment was repeated 4 times. *p < 0.05, **p < 0.01.

MAP kinase cascade is an additional signal transduction pathway downstream of TLR activation (reviewed in [37]). Therefore, we examined activation of the JNK and p38 MAP kinases to identify signaling events that might connect elevated p53 levels to increased cytokine production. The induction of JNK phosphorylation upon flagellin exposure was comparable between MCF7-vector and MCF7-p53i cells regardless of Nutlin-3 treatment (Figure 4A, upper panel). In contrast, flagellin-induced phosphorylation of p38 was dramatically increased in MCF7-vector cells treated with Nutlin-3 (Figure 4A, middle panel). This effect was entirely p53-dependent as it did not occur in MCF7-p53i cells.

To test whether enhanced activation of p38 by flagellin in the Nutlin-3 treated cells is responsible for increased cytokine production, the cells were exposed to the p38 kinase inhibitor SB203580 prior to flagellin. p38 inhibition selectively attenuated induction of 30 out of 208 Synergistic Target genes, as demonstrated by microarray analysis (Figure 4B-4C and Suppl. Table 1). This group of p38 responsive genes included *IL6, CCL22, CXCL10, SAA2, S100A8* and *S100A9*. However, there was no effect of SB203580 on expression of many other Synergistic Targets such as *IL8, PLAU* and *PLAUR* (Figure 4D, Suppl. Table 1). These results suggest different mechanisms for the p53-enhanced expression within the Synergistic Targets genes.

Since p38 can increase expression of several inflammation genes including IL6 and IL8 via transcript stabilization [38], we examined the transcript stability of IL6. The transcription inhibitor Actinomycin D was added at 1 h after addition of flagellin to the MCF7 cells, and transcript levels were followed for 60 min. In contrast to p21 mRNA levels, which remained stable, IL6 mRNA dropped considerably, demonstrating the labile nature of this transcript (Suppl. Figure 5). However, the transcript degradation was not affected by Nutlin-3 pretreatment, ruling out mRNA stabilization as a mechanism of IL6 induction in our system.

### PI3 kinase and STAT3 mediate increased transcription of a subset of Synergistic Target genes

**Figure 5:**
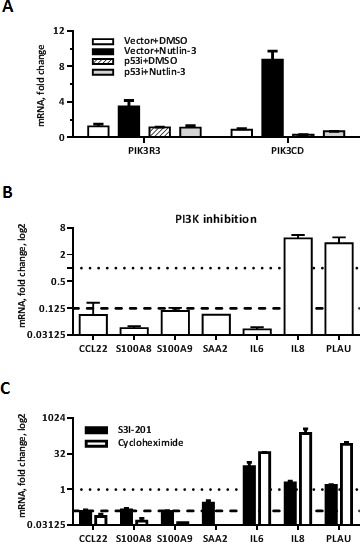
PI3 kinase and STAT3 mediate increased transcription of a subset of p38-dependent Synergistic Target genes **A.** Change in mRNA expression of PI3K subunits PIK3CD and PIK3R3 following 48 h of Nutlin-3 treatment in MCF7 cells. **B.**-**C.** MCF7-vector cells were treated with Nutlin-3 for 48 h. During the last 4 h the medium was replaced with DMSO/Nutlin-3 medium containing either 20 μM PI3K inhibitor LY294002 (B), 100 μM STAT3 inhibitor S3I-201 (C, black bars) or 25 μM translation inhibitor cycloheximide (C, white bars) for 60 min and then the cells were incubated with flagellin for 3 h. Cells were harvested and mRNA expression of indicated Synergistic Target genes was measured by qPCR. Presented are ratios of indicated genes expression in Nutlin-3 + flagellin-treated samples pretreated with corresponding inhibitor compared to expression in control samples treated with Nutlin-3 + flagellin only. The dotted lines indicate no change (*i.e.,* value of 1) and the dashed line indicates strong inhibition (value of 0.125). Presented are average changes in expression from 3 independent experiments.

Among the genes upregulated by Nutlin-3 in MCF7-vector cells, we identified two subunits of PI3 kinase, the regulatory subunit p55 gamma PIK3R3 and catalytic p110 delta subunit PIK3CD (Figure 5A). Since several studies [39, 40] suggested that TLR5 activation engages PI3K, we investigated a role for PI3K in the synergistic response using PI3K inhibitor LY294002. Treatment of cells with LY294002 reduced over 8-fold the p53/flagellin-dependent induction of several Synergistic Target genes including *CCL22, S100A8, S100A9, SAA2* and *IL6*, but not *IL8* and *PLAU* (Figure 5B).

Next, we sought to identify possible transcription factors that could mediate synergy between p53 and flagellin and integrate p38 and PI3K kinases activity. Jak-STAT and cytokine-cytokine receptor signaling pathways were enriched in a KEGG pathway analysis of the Synergistic Targets gene set (Table 1), and STAT3-dependent transcription was strongly activated in response to the flagellin analog CBLB502 in mouse liver [15]. Consistent with these findings, we found that specific inhibition of STAT3 by S3I-201 prevented the p53-dependent increase in induction of several Synergistic Targets including *CCL22, S100A8, S100A9* and *SAA2* without affecting the synergistic induction of *IL6, IL8* and *PLAU* (Figure 5C, black bars). Therefore, genes controlled by STAT3 overlap with PI3 kinase and p38 targets.

Activation of STAT3 by TLR signaling is a secondary event in response to PAMP exposure. Following TLR stimulation cytokines such as IL6, IL10, IL21 are produced and activate STAT3 via interaction with the corresponding cytokine receptors (reviewed in [41]). Consistent with this indirect mechanism, inhibition of protein synthesis by cycloheximide during activation of TLR5 by flagellin in Nutlin-3 pretreated MCF7-vector cells prevented the induction of STAT3-dependent genes (Figure 5C, white bars). Taken together, our results suggest that STAT3 mediates a secondary wave of Nutlin-3 enhanced secretion of the cytokines that are rapidly synthesized after TLR5 stimulation.

### Synergistic genes are elevated in breast cancer patients responsive to chemotherapy

**Figure 6:**
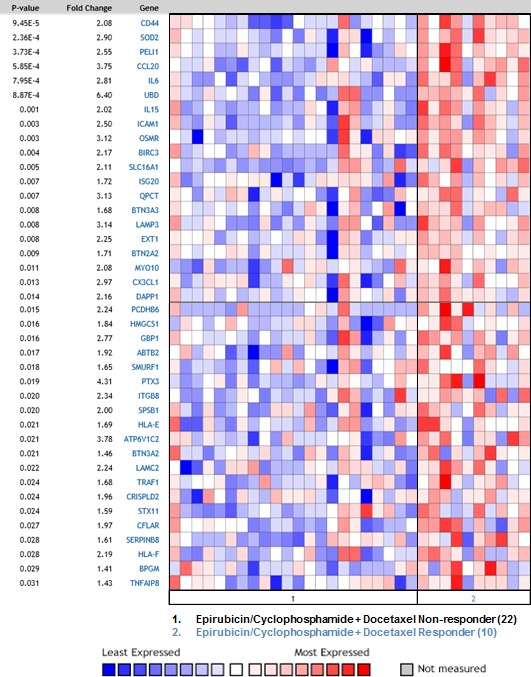
Synergistic genes are elevated in breast cancer patients responsive to chemotherapy Synergistic Target genes are significantly over-represented in the gene signature that was overexpressed in breast cancer patients responsive to epirubicine and cyclophosphamide, followed by docetaxel as compared to non-responders and correlated with pathological complete response (pCR) [43]. pCR was defined as complete disappearance of the tumor following neoadjuvant chemotherapy. The study included 10 responders and 22 non-responders. The Oncomine™ Platform (Life Technologies, Ann Arbor, MI) was used for analysis and visualization.

Along with its pro-tumorigenic effects, inflammation also induces the host anti-tumor immune response and can be used in cancer immunotherapy [42]. Since inflammation-related genes are enriched among Synergistic Targets we looked whether there is a connection to response to chemotherapy. The complete list of Synergistic Targets was compared to the precomputed molecular signatures in Oncomine (www.oncomine.org) database. This meta-analysis revealed that our list of 208 genes overlaps significantly with the gene signatures that were overexpressed in breast cancer patients responsive to neoadjuvant chemotherapy (NAC) and predicted pathological complete response (pCR). Table 3 describes five independent studies where significant overlap was found. The statistical significance of this overlap is indicated by a *p*-value. Figure 6 displays expression levels of 40 Synergistic Target genes, most significantly overexpressed in the tumors from responders in the study by Stickeler et al. [43]. The *p*-values in Figure 6 represent the significance of differential expression between responder and non-responder groups for each Synergistic Target gene. Lists of genes consisting molecular signature for each of five studies are presented in Suppl. Table 2. Our findings suggest that an increase in inflammatory response by a combination of p53 induction and TLR5 activation might be beneficial in breast cancer treatment. Therefore, in-vivo studies are warranted where TLR5 ligand is combined with traditional chemotherapy in order to induce an expression of these genes and potentially improve the treatment outcome. Our results suggest that such combination might be particularly effective in augmenting the immune response in tumors with wild-type p53, which will be activated by cytotoxic drugs.

**Table 3:** Synergistic genes are over-represented in patients responsive to chemotherapy

Cancer type	Treatment	Study size	*p*-value	Overlap	Ref.
Ductal Breast Carcinoma	fluorouracil, epirubicin, and cyclophosphamide	34+22	8.34E-7	42 genes	[56]
*Invasive Breast Carcinoma	docetaxel	5+3	6.76E-5	18 genes	[57]
Invasive Ductal Breast Carcinoma	fluorouracil, doxorubicin, and cyclophosphamide	76+7	9.48E-6	34 genes	[58]
Invasive Ductal Breast Carcinoma	paclitaxel and fluorouracil, doxorubicin, and cyclophosphamide	63+17	1.89E-7	11 genes	[58]
Invasive Breast Carcinoma	epirubicine and cyclophosphamide, followed by docetaxel	22+10	4.88E-12	53 genes	[43]
Breast Carcinoma	paclitaxel followed by fluorouracil, epirubicin, cyclophosphamide	88+27	3.10E-11	51 genes	[59]

208 Synergistic Target genes (Supplementary Table 1) were analyzed using Oncomine Research Premium Edition software to compare to gene signatures that were significantly overexpressed in patients responsive to neoadjuvant chemotherapy (NAC). These signatures predicted pathological complete response (pCR) in several independent studies. pCR was defined as complete disappearance of the tumor following chemotherapy. *In this study pCR was defined as >75% post-treatment decrease in tumor size. The gene expression was measured in core biopsies of the primary cancers before NAC. Study size consists of number of non-responders + responders patients involved. Overlap is a number of genes common between Synergistic Targets list and the gene signature predicting response to NAC in a specific study. The statistical significance of this overlap is indicated by a p-value.

## DISCUSSION

**Figure 7:**
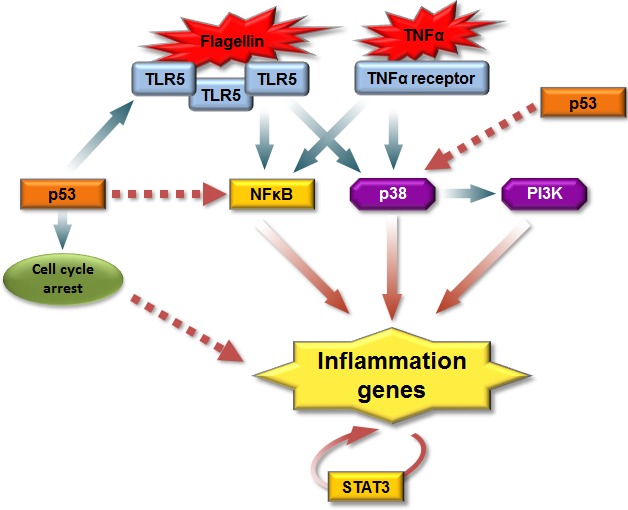
A model for p53 regulation of the receptor-mediated inflammatory response Upon stimulation by ligands, both TLR5 and TNFα receptor recruit adaptor molecules leading to activation of NFκB and mitogen-activated kinase (MAPK) signaling pathways, which result in distinct expression patterns of immune/inflammatory genes. Also PI3K/AKT pathway is required to initiate host immune response and p38 acts upstream of PI3K. p53 can enhance TLR5 inflammatory response through increase in the receptor levels, by direct enhancement of transcription or via increased activation of the aforementioned signaling pathways. Induction of cell cycle arrest due to activation of p53 can increase ligand-dependent TLR5 activity. Increased cytokine secretion generates a secondary wave of expression of inflammatory genes through the STAT3 pathway. Solid arrows stand for known interactions and dashed arrows stand for proposed interactions.

p53 is activated by several stress signaling pathways such as DNA damage, starvation, oxidative stress or oncogenes and integrates these signals into transcriptional response. Using the genome-wide expression analysis we established that induction of p53 protein can synergistically enhance the TLR5-induced gene expression program in both primary and cancer cells. In addition, we provide evidence for p53-mediated augmentation of signaling downstream of other extracellular ligands such as TNFα. The synergy was observed both for the situations where the levels of the corresponding receptor were induced by p53 (TLR5 in MCF7) or not (TNFα in MCF7; TLR5 in A549, HCT116, CD14+, CD3+ cells). Therefore, while p53-induced increase in TLR5 expression can contribute to synergy, our results clearly show that synergy involves additional factors/signaling pathways (Figure 7).

We found that potential transcription targets of NFκB were highly enriched among the synergistically induced genes and the p65 subunit of NFκB was required for their expression. Nevertheless, our results excluded the possibility that the synergy resulted from an enhancement of NFκB activity by p53 such as modulation of NFκB expression levels, nuclear translocation or chromatin binding. Except for BBC3 gene, there were no examples among the Synergistic Targets examined that binding of NFκB to its corresponding response element was increased in the presence of high levels of p53.

p53 primarily acts as a sequence-specific transcription factor. However, there are several examples showing that p53 can enhance transcription that is mediated by other transcription factors from their respective nearby binding sites. Recently, Lion *et al.* discovered that the combined activation of p53 and estrogen receptor α pathways induce expression of a unique set of genes beyond those induced by activation of only p53 or estrogen receptor [44]. Bisio *et al* have shown that combination of p53 and TNFα receptor activation resulted in synergistic gene expression [45]. Choy *et al* demonstrated that mir-21 expression depends on formation of p53/NFκB/STAT3 complex at NFκB response elements (REs) at the mir-21 promoter. In addition, several histone modifying enzymes including histone methyltransferases (PRMT1 and CARM1) and histone acetyltransferases (p300/CBP, pCAF, GCN5 and TIP60) are recruited to DNA in a p53-dependent fashion in the vicinity of p53 REs and thereby facilitate formation of pre-initiation complex and RNAPII-dependent transcription (see [46] for review). Our analysis shows that p53 can potentially bind at promoter areas of many Synergistic Targets. Therefore, one possible mechanism by which p53 can enhance transcription of these genes could involve p53 assisting in the recruitment of transcriptional machinery to these promoters, thereby priming them for increased transcription.

Increased p38 activation represents another mechanism that mediates transcriptional synergism between p53 and TLR5 pathways. Although activation of p53 by p38 was described previously [47, 48], our study is the first to demonstrate a reciprocal effect where induction of p53 increases p38 activation capacity. We also show that p53 induces expression of two PI3K subunits, PIK3R3 and PIK3CD and that an increased transcription of a subset of Synergistic Target genes is prevented by inhibition of PI3 kinase. These results suggest that activation of PI3K activity by p53 might play a role in the synergy mechanism. Additional studies are necessary to test this hypothesis. The overlap between p38-dependent and PI3K-dependent Synergistic Targets suggests a crosstalk between these pathways in the context of p53/TLR synergy in the innate immune responses. Indeed, McGuire and colleagues recently demonstrated that both PI3K/Akt and p38 are required to initiate host immune responses in bone marrow-derived macrophages and that p38 acts upstream of AKT [49].

We identify STAT3 as an additional signal transduction pathway involved in p53/TLR5 synergy. Our experiments with protein synthesis inhibitor, taken together with previously reported studies suggest that this pathway is responsible for a second wave of cytokines which can be activated by newly synthesized cytokines during the early stage of TLR5 activation.

There is a well-established link between the p38, NFkB, PI3K and STAT3 pathways, inflammation, resistance to apoptosis and cancer [50-52]. Therefore, upregulation of downstream targets of these signaling pathways by p53 induction might potentially contribute to cancer progression. However, recent studies demonstrated that systemic activation of TLR5 in several syngeneic tumor models decreased tumor burden while significantly improving normal cell survival [15]. This effect was mediated by natural killer cells rather than by direct killing of tumor cells by TLR5 agonist [53]. In agreement, we found that synergistic genes are over-represented in patients responsive to chemotherapy, possibly modifying antitumor response. Similarly, Ji et al demonstrated that high expression of immune-related genes was favorably correlated with clinical response to cancer immunotherapy [54]. In addition, it was shown that activation of TLR5 by its agonist Entolimod reduces hematopoietic and gastrointestinal toxicity of 5- fluorouracil in mice without reducing its antitumor activity [55]. Therefore, we propose that the combinatorial activation of p53 and TLR5 pathways could affect tumor microenvironment, increase anti-tumor immunity by recruitment of immune cells to the tumor site and thereby improve cancer treatment. That being the case, TLR5 ligands should be evaluated as adjuvants to standard therapies that involve induction of p53.

## MATERIALS AND METHODS

### Reagents and antibodies

Nutlin-3 (10 μM, DMSO), GW8510 (4 μM, DMSO), Flavopiridol (300 nM, DMSO), Actinomycin D (10 μg/ml, DMSO) and S3I-201 (100 μM, DMSO) were from Sigma (St. Louis, MO). Flagellin (500 ng/ml) and TNFα (10 ng/ml) were from Invivogen (San Diego, CA), LY294002 (20 μM, DMSO) from Cayman (Ann Arbor, MI), SB203580 (10 μM, DMSO) and cycloheximide (25 μM, DMSO) were from EMD Chemicals (La Jolla, CA). The working concentrations of each chemical and solvent used to prepare stock solution are indicated in parentheses.

The primary antibodies were as follows: against p53 (DO-1) and actin (R-19), Santa Cruz Biotechnology (Santa Cruz, CA); p21 (SXM30), BD Biosciences Pharmigen (San Diego, CA); pp38 (#9211), p38 (#9212), pJNK (#9251), p-IκBα (#2859), and IκBα (#4814), Cell Signaling Technology (Danvers, MA); p65 (ab7970) and TBP (ab818 ), Abcam (Cambridge, MA); and p105/p50 (#1559-1), Epitomics (Burlingame, CA).

### Cell culture

Human lung adenocarcinoma cells A549 and ovary adenocarcinoma Caov3 were obtained from ATCC (Manassas, VA). Human breast adenocarcinoma cells MCF7 stably expressing shRNA to p53 from the pSUPER vector, designated as “MCF7-p53i”, or carrying pSUPER vector as a control (“MCF7-vec”) were kindly provided by Dr. R. Agami [26] and the cell line identity was verified by short tandem repeat profiling (DDC, Fairfield, OH). MCF7 and A549 cells were cultured in RPMI-1640 medium supplemented with 10% heat inactivated FBS for MCF7 and FBS for A549 and 50 U/ml of penicillin, 50 μg/ml of streptomycin (Invitrogen, Carlsbad, CA). Human colon carcinoma HCT116 p53+/+ and its isogenic derivative HCT116 p53−/− were provided by Dr. B. Vogelstein (The Johns Hopkins Kimmel Cancer Center, Baltimore, MD). HCT116 and Caov3 cells were cultivated in McCoy's and DMEM, respectively, supplemented with 10% FBS and penicillin/streptomycin. All cells were maintained at 37°C with 5% CO_2_.

### Primary cell isolation

Primary cells isolation was previously described [3]. The protocol was approved by Institutional Board Review IRB#10-E-0063. Cells were maintained in RPMI supplemented with 10% FBS, no antibiotics.

### Cell proliferation assay

For cell proliferation assays, cells were seeded at 2000 cells/well of a 96-well plate tray. At the end of indicated treatments the culture medium was replaced with 100 μl RPMI media containing 1% heat-inactivated FBS and penicillin/streptomycin and 20 μl of CellTiter 96® AQueous One Solution Cell Proliferation reagent. Cells were incubated at 37°C with 5% CO2 for 2 h and the color was measured with Synergy 2 Multi-Mode Microplate Reader (BioTek, Winooski, VT).

### Western blot analysis, subcellular fractionation and antibodies

Whole cell protein extracts were prepared using SDS lysis buffer (2% SDS, 50 mM Tris–HCl, pH 7.6), then sonicated for 15 cycles of 30 seconds on, 30 seconds off at high power setting using a Bioruptor sonicator (Diagenode, Denville, NJ) and centrifuged to obtain the supernatant. Where indicated, cytoplasmic and nuclear protein fractions were obtained using NE-PER protein extraction kit (Thermo Fisher Scientific, Cincinnati, OH) following the manufacturer's recommendations. Protein concentration was quantified using a BCA protein assay kit (Thermo Fisher Scientific). Equal amounts of protein (15-35 μg) were subjected to Western blot analysis as previously described [2].

### Transfections, siRNA and luciferase assays

Short interfering RNAs to p65 were purchased from Dharmacon and transfected at 100 nM using Dharmafect 1 (Thermo Fisher Scientific) per manufacturer's protocol at 6 μl of the reagent/well of 6-well plate.

For luciferase assays, MCF7-vector cells were seeded at 100,000 cells/well in 12-well plates and co-transfected with pNFκB-Luc vector (240 ng/well; Clontech, Mountain View, CA) and pRL-TK vector (10 ng/well; Promega, Milwaukee, WI) as a transfection efficiency control using FuGENE 6 reagent (Roche Molecular Biochemicals, Indianapolis, IN). Luciferase activity was measured using Dual Glo luciferase assay (Promega) with a Synergy 2 Microplate Reader.

### RNA purification, reverse transcription and gene expression profiling by qPCR and microarrays

RNA purification, reverse transcription and gene expression profiling by qPCR were previously described [2]. Gene expression analysis was conducted using Affymetrix Human Genome U133 Plus 2.0 GeneChip® arrays (Affymetrix, Santa Clara, CA). For clustering analysis the RMA-normalized file was filtered for SD>0.7 resulting in 1846 differentially expressed probes. Following gene centering and normalization, genes were clustered using average linkage hierarchical clustering with uncentered correlation similarity metrics as implemented by Cluster 3 software (http://bonsai.hgc.jp/~mdehoon/software/cluster/software.htm). Clustering results were visualized using Java TreeView (http://jtreeview.sourceforge.net). Gene lists were analyzed for enriched functional categories and transcription factor binding sites using GATHER interface [27]. Transcription factor binding sites were analyzed within the sequence 1200 bases upstream and 200 bases downstream of the annotated transcription start site. Synergistic Targets were defined as genes with expression induced greater that 2-fold by a combination of Nutlin-3 and flagellin compared with expression induced by each drug separately and synergy factor >1.5 (Synergy factor is an expression in combined treatment divided by the product of expressions upon single treatments) in MCF7-vector cells and the expression upon combined treatment in MCF7-vector cells 2-fold higher than in similarly treated MCF7-p53i cells.

### Chromatin immunoprecipitation (ChIP)

ChIP assays were performed as previously described [28]. Real-time PCR and melting curve analysis were performed in triplicates using the SYBR® Green (Life Technologies) master mix. Enrichment of specific targets was calculated as the fraction of Input (%) of DNA area of interest recovery in p65 immunoprecipitated samples or in unspecific IgG control samples. Primers for the NFκB response elements

PUMA: 5′ – CCCACAGTTTGGAAAACACCA – 3′,

5′ – CCCCGATTATTCATTGTCCCT – 3′

IL8: 5′ – GGGCCATCAGTTGCAAATC – 3′,

5′ – TTCCTTCCGGTGGTTTCTTC – 3′

Primers for the p53 response elements

IL6: 5′ – TCTCTTTGTAAAACTTCGT GCAT GA – 3′,

5′ – GATTGTGCAATGTGACGTCCTT – 3′

CCL20: 5′ – TCAAGGCTTGTTAGTTTTGT TGATCT – 3′,

5′ – AACTAGAGAAAAGCAGAGCAAGCTAAA – 3′

PIK3R3: 5′ – ATGTACCGGGATTCTGTCAAGTG – 3′,

5′ – CGGATCGTACCATTCTGATTGAG – 3′

CSF1: 5′ – GAGGAGTGGGCGCTTCTG – 3′,

5′ – CCCAAACAGCCACCCAAGT – 3′

### p65 *in vitro* binding assay

5 μg of total protein was used to measure p65 *in vitro* binding to immobilized oligonucleotides containing the NFkB consensus binding site (TransAM p65, Active Motif, Carlsbad, CA) according to manufacturer's instructions.

## SUPPLEEMENTARY MATERIAL FIGURES AND TABLE




