# Supplementary material for: p53 amplifies Toll-like receptor 5 response in human primary and cancer cells through interaction with multiple signal transduction pathways
Source: Oncotarget. 2015 Jun 10;6(19):16963–80. doi: 10.18632/oncotarget.4435 (PMC4627285; doi:10.18632/oncotarget.4435)
Supplement: Supplementary file 1 [file oncotarget-06-16963-s001.pdf]

p53 amplifies Toll-like receptor 5 response in human primary and cancer cells through interaction with multiple signal transduction pathways

Supplementary Material

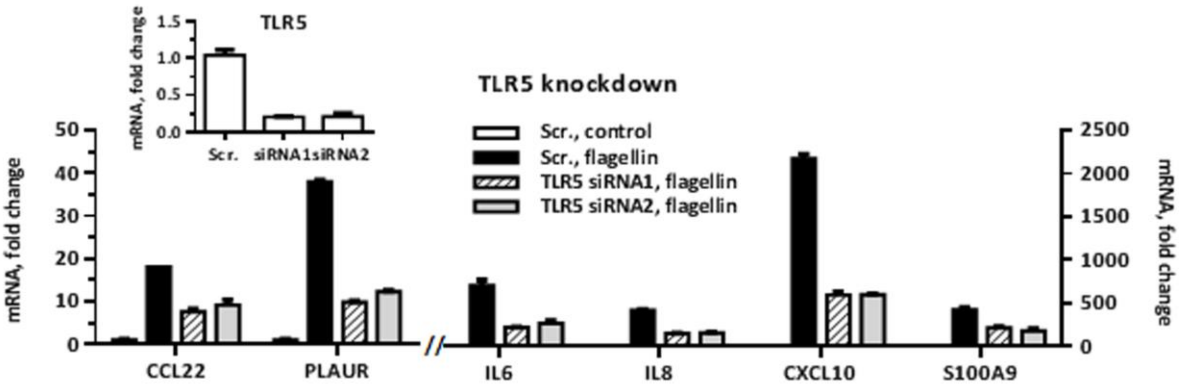

Supplementary Figure 1: Synergistic increase in gene expression is TLR5-dependent. MCF7-vector cells were transfected with TLR5 siRNA or scrambled control oligos (Dharmacon). 24 h later the cells were incubated with Nutlin-3 for additional 48 h and then exposed to flagellin for 3 h. Presented are fold-changes in mRNA expression for the indicated Synergistic Target genes.

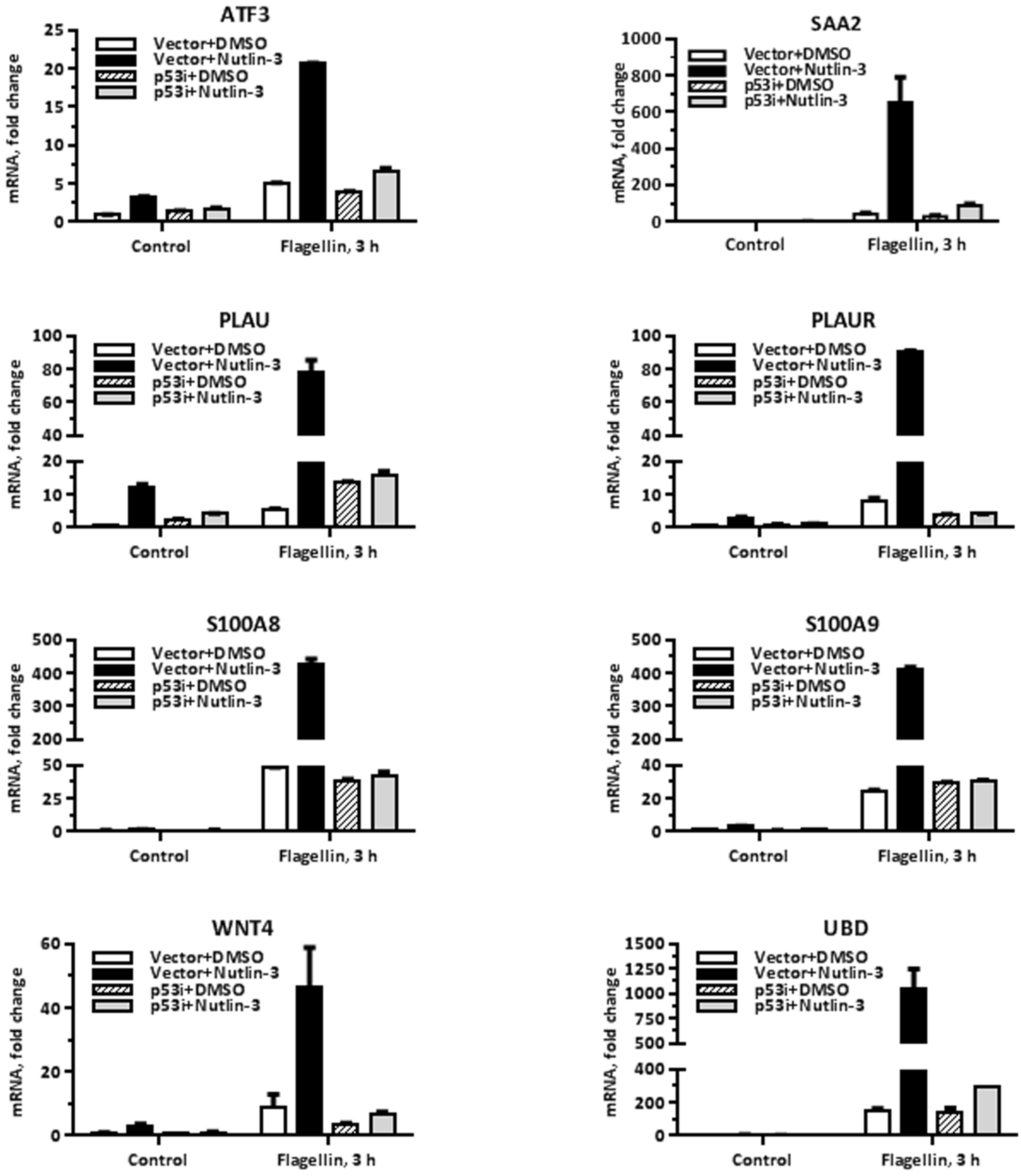

Supplementary Figure 2. Validation of microarray results. MCF7-vector or MCF7-p53i cells were incubated with Nutlin-3 or DMSO for 48 h. During the last 3 h the medium was replaced with DMSO/Nutlin-3 medium containing 500 ng/ml flagellin for 3 h. The mRNA was purified and expression of several genes identified earlier as Synergistic Targets (see Supplemental Table 1) was assessed using pre-designed real time PCR assays. Shown is a

representative examples of experiments repeated 3-6 times. The bars indicate range for PCR replicates.

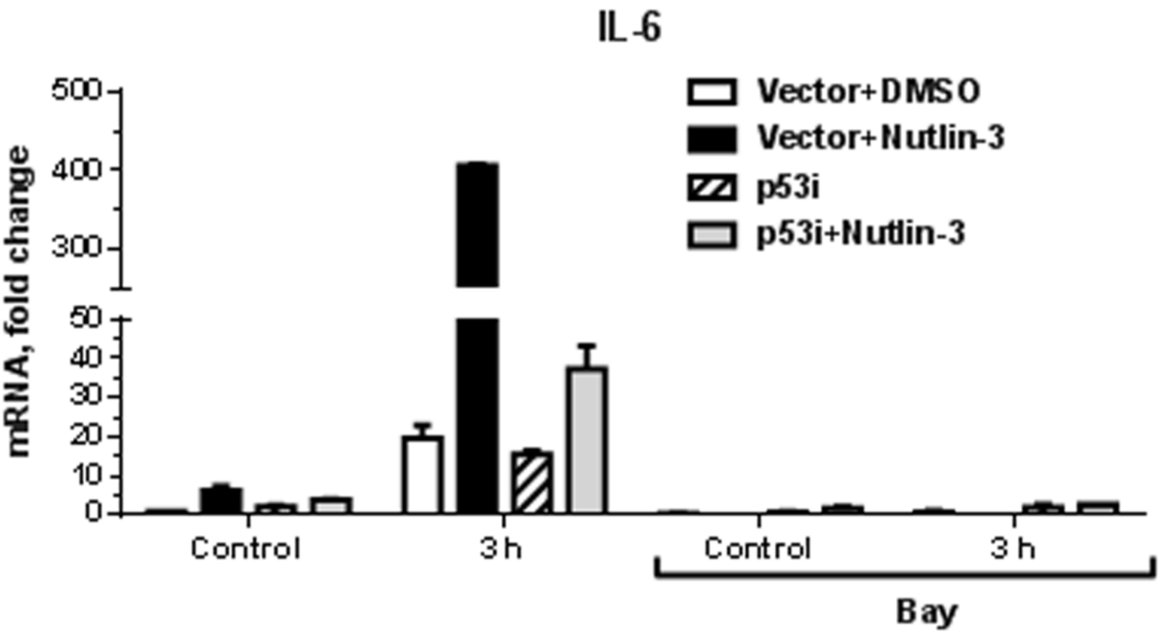

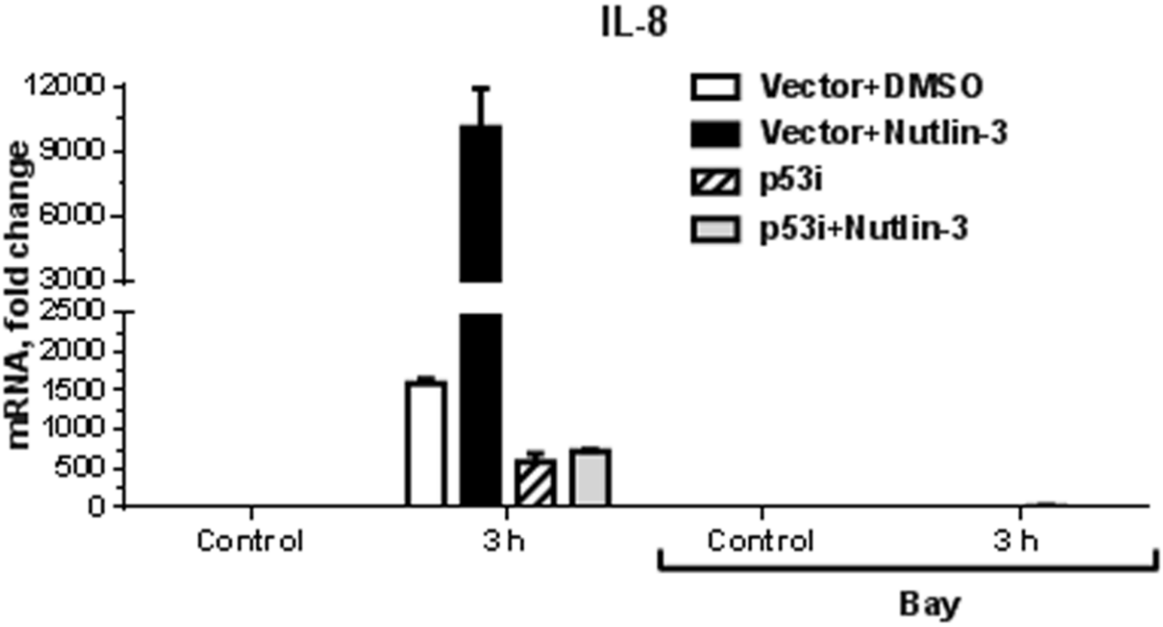

Supplementary Figure 3: Pan-inhibition of NFκB prevents induction of IL6 and IL8. MCF7-vector or MCF7-p53i cells were incubated with Nutlin-3 or DMSO for 48 h. During the last 4 h the medium was replaced with DMSO/Nutlin-3 medium containing pan-inhibitor of NFκB activity Bay117082 (50 µM) and after one hour flagellin was added to the cells for 3

h. The mRNA was purified and expression of several genes identified earlier as Synergistic Targets (see Supplemental Table 1) was assessed using pre-designed real time PCR assays. Shown is a representative examples of experiments repeated twice. The bars indicate range for PCR replicates.

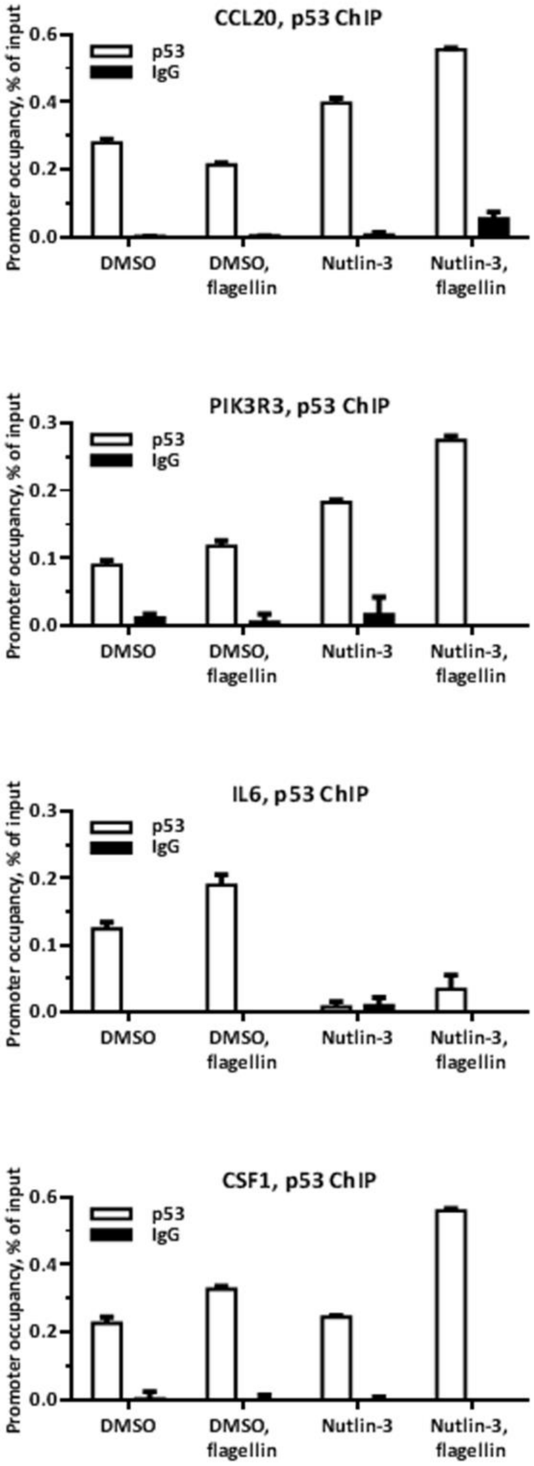

Supplementary Figure 4: Binding of p53 to the promoters of Synergistic Targets. MCF7-vector or MCF7-p53i cells were treated with Nutlin-3 or DMSO for 48 h and then incubated with flagellin for 2 h. p53 occupancy at CCL20, IL6 and PIK3R3 promoters was assessed by ChIP-qPCR.

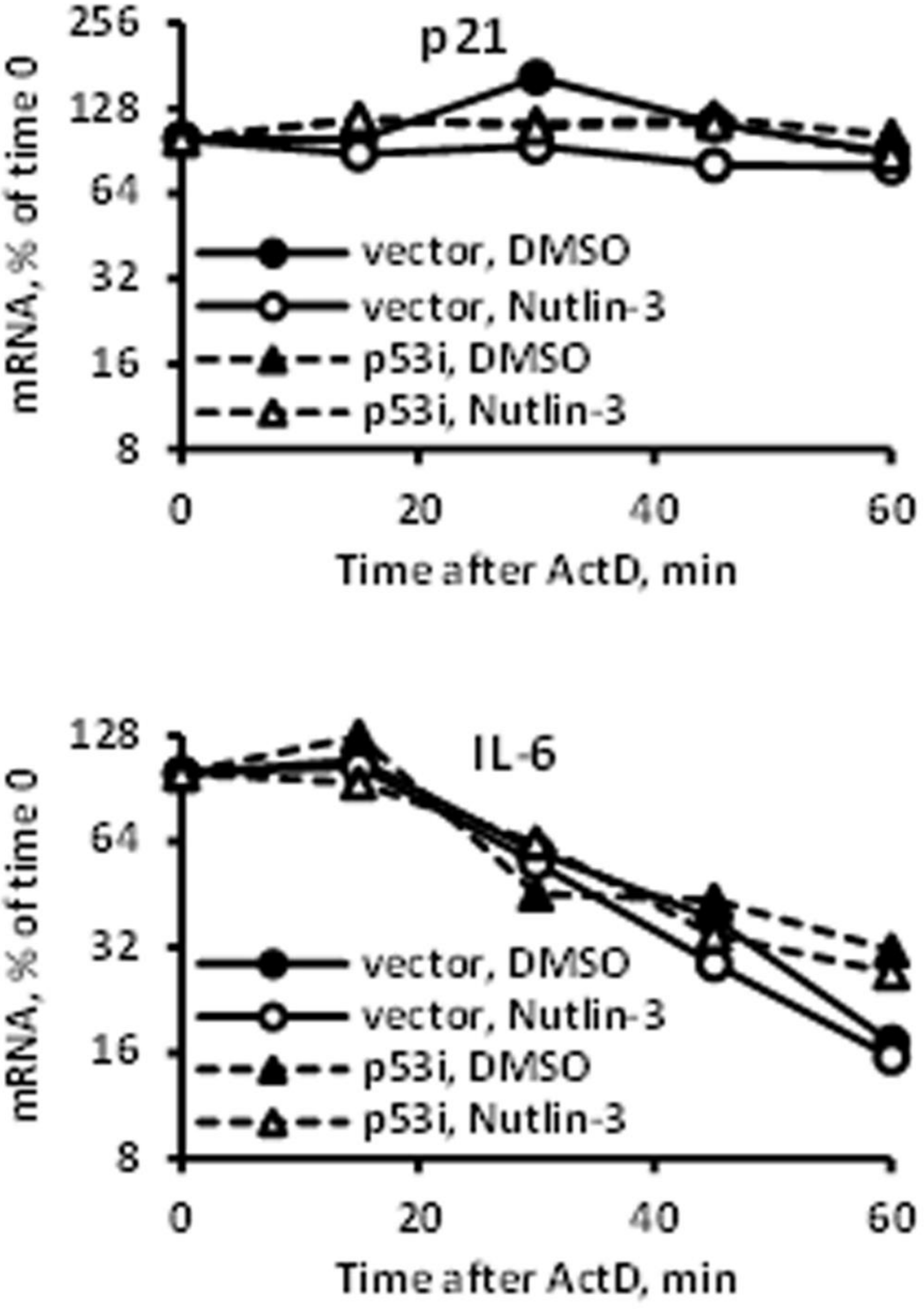

Supplementary Figure 5: Transcript stability of Synergistic Targets. MCF7-vector or MCF7-p53i cells were incubated with Nutlin-3 or DMSO for 48 h and then the medium was replaced with DMSO/Nutlin-3 medium containing flagellin. After 1 h of flagellin incubation Actinomycin D (10 μg/ml) was added to the medium (indicated as time 0 in the graphs). Cells were harvested at the indicated times and mRNA was purified. The mRNA levels are

presented as percent of expression at time 0, namely, just before Actinomycin D addition. Shown is a representative experiment that was repeated twice. Bars indicate range for PCR replicates.
